# Optimal Sensor Location Design for Reliable Fault Detection in Presence of False Alarms

**DOI:** 10.3390/s91108579

**Published:** 2009-10-27

**Authors:** Fan Yang, Deyun Xiao, Sirish L. Shah

**Affiliations:** 1 Department of Automation, Tsinghua University, Beijing 100084, China; E-Mail: yangfan@tsinghua.edu.cn (F.Y.); 2 Department of Chemical and Materials Engineering, University of Alberta, Edmonton, T6G 2G6, AB, Canada; E-Mail: slshah@ualberta.ca (S.L.S.)

**Keywords:** fault detection, directed graph, reliability, false alarm, missed alarm

## Abstract

To improve fault detection reliability, sensor location should be designed according to an optimization criterion with constraints imposed by issues of detectability and identifiability. Reliability requires the minimization of undetectability and false alarm probability due to random factors on sensor readings, which is not only related with sensor readings but also affected by fault propagation. This paper introduces the reliability criteria expression based on the missed/false alarm probability of each sensor and system topology or connectivity derived from the directed graph. The algorithm for the optimization problem is presented as a heuristic procedure. Finally, a boiler system is illustrated using the proposed method.

## 1. Introduction

Fault detection plays a necessary and important role in large-scale industrial systems for safety issues. Its basis and data source is the measurements from sensors. Measurement technology and sensor quality has progressed significantly in the past several decades, but the problem still exists because not all process variables of concern can be measured due to economic and technical limitations, and the reliability of sensors cannot be assured. In large-scale systems, the components are interconnected and so the variables are correlated, which constitutes information on system topology with causality. After a fault occurs, it not only shows up as local phenomenon but also propagates to some other components or variables. Hence we should consider the sensor location problem to find the root cause of the fault origin and type from the viewpoint of the whole system.

In order to measure the fault detection quality related to sensor location, some criteria are defined in Kawabata *et al.*'s paper [1]. Firstly, all the faults should be detected when they occur. Secondly, different faults should be identified from each other so that one can differentiate them based on the sensor readings. The criteria of detectability and identifiability are basic requirements for fault detection [2]. In this reference all the sensors are assumed to be effective, that is, they show exactly whether the process variables are normal or abnormal.

In engineering practice, sensors may often be faulty, meaning that they may fail to give adequate readings. For example, the reading may remain unchanged when the true value should be a deviation, which is called a missed alarm; or the sensor may give an alarm for a normal operation state, known as a false alarm. We should therefore allow for some redundancy in sensors in case of failures. More commonly, the measurements may show these two kinds of sensor faults because of the choice of the threshold. Often due to noise there are no real sensor faults but deviations due to measurement noise, which is inevitable. If the threshold setting is strict in order to suppress the missed alarm probability, the reading will be sensitive to random noise and temporary deviations, resulting in a high probability of false alarm. If we relax the threshold and accept larger region to be considered as normal, then the number of false alarms will decrease with more missed alarms. Therefore, missed alarms and false alarms are two aspects of reliability and we have to make a trade-off between them. This can be clearly illustrated via a receiver operating characteristics (ROC) curve [3,4]. Sensors in this present paper also include soft sensors that measure some specific variables by soft sensing techniques [5].

With increasing complexity in process industrial systems, traditional mathematical models are difficult to obtain. Hence, graph-based models are proposed in the modeling analysis. Based on the signed directed graph (SDG) model, Raghuraj, *et al.* [2] have discussed the problems of detectability and identifiability in sensor location and presented the corresponding algorithm for locating each sensor. These methods are based on a certain static SDG and omit the propagation time of faults. Yang and Xiao [6] introduced the propagation time in SDGs, based on which the problem has been defined and solved, and some applicable rules have been presented to obtain a reasonable sensor location. Bhushan and Rengaswamy [7] studied the reliability problem of fault detection and proposed some algorithms to choose sensor location to improve reliability. Bhushan *et al.* [8] also studied the robustness of the network to uncertainties/errors in the underlying model and probability data. However, only missed alarm probability has been considered in their work. On the other hand, the false alarm probability should also have been taken into account because adding sensors increases the number of false alarms that is undesirable. The reliability optimization problem of false alarms is discussed in this paper as a complementary criterion to the optimization problem of undetectability.

This paper is structured as follows: The criteria of fault detection, especially the reliability criterion regarding false and missed alarms in sensor readings, are presented in Section 2. Section 3 explains how to use graph theory to obtain the reachability measure between faults and process variables measured by sensors, which is needed in the optimization criteria. In Section 4, the optimization algorithm for the sensor location is proposed to improve the reliability of fault detection, followed by a case study to illustrate the application in Section 5. Finally some concluding remarks are given in the last section.

## 2. Sensor Location Criteria for Fault Detection

There are basic criteria that should be met under all fault detection issues, and also optimization criteria in consideration of faulty sensors or unreliability of sensor measurements.

### 2.1. Detectability and Identifiability

The nodes in the SDG are classified into two types–variables and fault origin actors, which are denoted as *n_i_*'s and *f_j_*'s respectively. When a fault occurs, it is propagated along consistent paths as designed in the SDG convention.

#### Definition 1

Starting from the fault node *f*, the set of nodes affected by *f* is *R*(*f*) = {*m* : ∃*l* (*f* ↦ *m*)} where *l*(*f* ↦ *m*) means a path from fault *f* to node *m*. If *n* ∈*R*(*f*), then we say that node *n* is reachable from fault *f*.

Regarding detectability, each fault should be detected by at least one sensor. The definition of detectability appears below:

#### Definition 2

If there exists at least one sensor placed in the nodes of *R*(*f*) (measuring the corresponding variables), then we say that fault *f* is detectable.

Because the propagation time is ignored here, only leaf nodes are needed to consider whether or not to place sensors [2]. Then we have the following theorem from Yang and Xiao [6].

#### Theorem 1

Based on the SDG, disregarding the cases that some variables cannot be measured, sensors need to be placed only on the leaf nodes.

##### Proof

According to the weak connection condition (i.e., the corresponding undirected graph is connected), each fault origin has at least one path to the leaf nodes, thus placing sensors on the leaf nodes can meet the detectability criterion. Assume that a sensor location with *n* nodes meets the detectability criterion, we can then search an arbitrary path from each node to the corresponding leaf node and place a sensor on this node, then this new scheme can also meet the criteria, and the number of sensors is no more than *n* (less than *n* if some nodes correspond to the same leaf node). Q.E.D.

Different faults have different behaviors. Represented in the SDG, the reachable nodes from the faults are different. So we must place sensors on these different nodes to identify the different faults. The definition of identifiable faults as noted in [2] appears below:

#### Definition 3

If there exist at least one sensor on the nodes of *R* (*f*_1_) (measuring corresponding variables), and these sensor nodes are not within the nodes of *R* (*f*_2_), in other words, if there are sensors in the nodes of *I* (*f*_1_, *f*_2_) = *R*(*f*_1_) ∪ *R*(*f*_2_) − *R*(*f*_1_) ∩ *R*(*f*_2_), then we say that faults *f*_1_ and *f*_2_ are identifiable.

Detectability and identifiability are two independent concepts. A fault can be detectable, but it may not be identifiable. On the other hand, identifiability does not imply detectability in general, because we can place only one sensor to identify them. But usually we assume that only when the faults are detectable, can they be considered for identifiability. Thus the identifiability criterion is stronger.

It should be noted that the signs of the nodes and branches can help identify different faults because some sensors are not only able to activate the alarm, but also indicate the direction of the departure from the normal values. For this case, we can split a node into two, one may show a higher deviation, and the other may show a lower deviation [9]. Then the above definition can be applied.

### 2.2. Reliability with Respect to Sensor False Alarms and Missed Alarms

**Figure 1.**
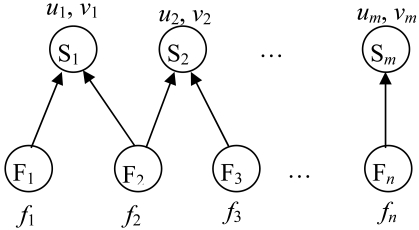
Bipartite graph to show the relations between faults and sensors.

Detectability and identifiability are necessary conditions for fault detection. However sensor readings are not always reliable, which affects the reliability of fault detection. Let F*_i_*s (*i* = 1, 2, …, *n*) and S*_j_*s (*j* = 1,2,…,*m*) denote system faults and process variables measured by sensors individually. They can be shown as a bipartite graph with all the arcs directed from the fault set to the process variable set as shown in Figure 1 [7]. Based on the detectablity criterion, there should be at least one arc departing from every fault node, and based on the identifiability criterion, the connected sensor nodes of different fault nodes should be different. The fault occurrence probabilities of the fault F*_i_* is *f_i_*, while the sensor missed alarm probability and false alarm probability of variable S*_j_* is *u_j_* and *v_j_*. The influence relation from fault F*_i_* to sensor S*_j_* is denoted by reachability *d_ij_* (0 or 1) where 1 means reachable and 0 means unreachable. Because of the causal relations between process variables, the reachability includes direct and indirect influences.

**Figure 2.**
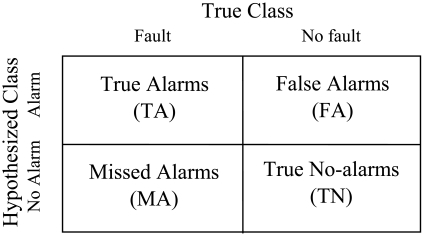
Confusion matrix to show the terminology of missed alarms and false alarms.

As shown in Figure 2, the confusion matrix reflects the true/false classification of alarms [3]. The entries in the matrix are the number of true alarms (TA), false alarms (FA), missed alarms (MA) and true no-alarms (TN) that means no alarm occurs under normal situation.

These numbers can be obtained by experiments. The missed alarm probability of sensor S*_j_* is *u_j_* which can be calculated by MA/(TA+MA), and false alarm probability of *v_j_* can be calculated by FA/(FA+TN). These probabilities are determined by the sensor quality and the threshold selection.

For each fault F*_i_*, we should minimize its probability of not being detected. Because it is propagated to many other variables whose sensors can also detect it, the undetectability of F*_i_* occurs only when all the variables miss alarms. In addition, the redundant sensors on the same variables are also helpful for the improvement. We define the undetectability probability [7] of F*_i_* as:

(1) $$U_{i}=f_{i}\left(\prod_{j=1}^{m}{\left(u_{j}\right)}^{d_{\text{ij}}x_{j}}\right)$$

where *x_j_* is the integer number of sensors placed on the variable *j*. If there is no sensor on variable *j*, *x_j_* is zero. Obviously, when *x_j_* with the corresponding nonzero *d_ij_* increases, *U_i_* decreases. So adding sensors will increase the reliability.

On the other hand, we need to be concerned about the false alarm problem. For the variable S*_j_*, adding a sensor with false alarm probability *v_j_* will be accompanied with the increase of the following false alarm probability:

(2) $$V_{j}=v_{j}(\prod_{i=1}^{n}{\left(1-f_{i}\right)}^{d_{\text{ij}}})$$

which means that the sensor reading will lead to an alarm even though no faults occur.

The calculations of missed alarms and false alarms are dual problems, in which adding sensors will reduce the undetectability whilst increasing the false alarm probability. Here the false alarm probability reflects the influence of a sensor's false alarm on the whole system.

## 3. Reachability Information Derived from Diagraphs

When a fault occurs, it will be measured not only by the adjacent sensors directly, but also by the influenced sensors due to propagation between variables. In order to describe the propagation, the SDG has been proposed as a qualitative model which uses nodes and arcs to denote the variables and their causal relations [10,11]. Along the consistent paths in the SDG, one can easily find how a fault is propagated in the system. Thus the reachability matrix mentioned in above section can be obtained from SDG. Here the signs are ignored and only the adjacency is taken into account.

Signed adjacency matrix is an equivalent expression of the SDG, whose elements ‘0’s, ‘+1’s or ‘−1’s correspond to the arc signs in SDG. For the (*i, j*)th element, ‘+1’ means there is a direct positive causality from node *i* to node *j*. Similarly, ‘−1’ means negative causality, and ‘0’ means no direct causality. Note that the matrix is asymmetric because the arcs are directional. In this paper, the sign of the element is of no use because we are merely concerned if there is direct relation between the two variables. Thus ‘+1’ and ‘−1’ are regarded as the same. Then we obtain the adjacency matrix with elements ‘0’ or ‘1’.

Given the adjacency matrix ***X***, the (*i, j*)th elements in ***X**^k^* give the number of *k*-step paths from node *i* to node *j*. And the summation of ***X**^k^* with *k* from 1 to *n* (dimension of ***X***) shows the reachability between every two nodes. The reachability matrix is then defined as [12]:

(3) $$\text{R}={\left(\sum_{i=1}^{n}{\text{X}}^{k}\right)}^{\#}$$

where the operator ‘#’ is the Boolean equivalent for all the elements *a*(*i,j*)s, which means there is at least a path in the corresponding SDG:

(4) $$a^{\#}(i,j)=\{\begin{array}{c} 0,\;\text{if}\;a(i,j)=0\\ 1,\;\text{if}\;a(i,j)\neq 0 \end{array}$$

The diagonal elements in ***R*** are designated as 1's because the fault on a sensor itself will show in the reading without propagating through other variables.

The reachablity matrix can also be obtained by graph traversal instead of matrix computations. The depth first search method can be used to find the paths. The graph traversal method has many advantages compared with the matrix computation method. First, the paths can be obtained in addition to the reachability matrix, which is intuitive and may help for the fault propagation and other analysis [13]. Secondly, when the structure of SDG changes slightly and only some local nodes or arcs are added or removed, we do not need to compute the matrix once again but only need to analyze the affected paths. This case can often be met when sensor location is updated based on a sensor location algorithm.

The reachability matrix is also a probabilistic value because the connectivity may be broken for some reasons. This random factor, however, is quite small compared with the measurements, so it is ignored and so the reachability is regarded as a binary value.

## 4. Algorithms

The two criteria, detectability and identifiability should be met at first when deciding the sensor location. Yang and Xiao [6] proposed an algorithm and some useful rules to solve this problem in consideration of the propagation time, which is a stricter requirement than that mentioned above. The sensor location obtained has the minimum number of sensors required for fault detection. Since the increase of sensors will not destroy these criteria, the following optimization algorithm should be based on this location and try to find the crucial variables for placing additional sensors.

In the trade-off between false alarms and missed alarms, missed alarms are often considered to be more important because we do not want to lose a real fault. Thus the algorithm handles this criterion first. Meanwhile, we hope that the false alarm probabilities will be as small as possible, so we integrate the treatment of false alarms into the whole algorithm.

If we consider all the faults, then we want to minimize the total undetectability probabilities for all the faults, each one of which is a probability that no sensors indicate the alarm for the corresponding fault. According to the assumption of origin of a single fault [10], the undetectability probability of the system is the summation of each fault. This assumption makes sense because probability of the simultaneous occurrence of more than one fault is extremely small. Therefore we have the following optimization problem:

(5) $$\underset{x_{j}}{\min}\left[\sum_{i=1}^{n}U_{i}\right]$$

This optimization problem cannot be solved analytically for the following reasons. First, this problem does not have a continuous solution space; instead it is an integer programming problem. Thus we should update the solution (*x_j_*, *j*=1,…,*m*) by changing the integer combination, where *x_j_* is the number of sensors placed on variable *j*. Secondly, the problem has inequality constraints. For example, placing a sensor on a variable is at a cost, and the total cost should be limited within a range, so we have:

(6) $$\sum_{j=1}^{m}c_{j}x_{j}\leq C_{0}$$

where *c_j_* is the cost of placing a sensor on variable *j*, and *C*_0_ is the cost limit. In addition, there may be other constraints due to technical or other reasons. Sometimes we have more constraints such as the number limit of sensors. Thirdly, the initial value of the problem is obtained according to the criteria of detectability and identifiability, and the *x_j_*'s should not be negative, which can be regarded as another constraint. This algorithm is then used to reduce the undetectability probability by adding sensors at critical location. Of course this problem can be solved by classical integer programming algorithms such as branch and bound method. However in real applications, what one needs most is to improve the result at the least expense. Thus the problem is proposed to be solved by an iterative algorithm, and within each step we should only add 1 to one of the *x_j_*s and then check the constraints. This is a heuristic algorithm.

On the other hand, the probability of false alarm of the system is a product of probability that no faults have occurred and the probability that at least one of the sensors indicates a false alarm. Then the problem can be expressed as:

(7) $$\underset{x_{j}}{\min}\left(\prod_{i=1}^{n}\left(1-f_{i}\right)\right)\left(1-\prod_{j=1}^{m}{\left(1-v_{j}\right)}^{x_{j}}\right)$$

We introduce a similar assumption that at most one sensor will indicate a false alarm. This assumption is reasonable when the false alarm probability is small. Thus the false alarm problem can also be formalized as an integer optimization problem:

(8) $$\underset{x_{j}}{\min}\left(\sum_{j=1}^{m}x_{j}V_{j}\right)$$

This expression is an approximation in order to simplify the computation. When adding a sensor, we can just add a *V_j_*, otherwise we have to compute it by using Equation 7. This problem is accompanied with the undetectability optimization problem and is less important for most cases. Thus we do not take it as an individual problem but as a complement to the above problem expressed by the following formulation:

(9) $$\underset{x_{j}}{\min}[\sum_{i=1}^{n}U_{i}+\alpha \sum_{i=1}^{m}x_{j}V_{j}]$$

where α is a constant coefficient.

When trying to reduce the undetectability by adding a sensor, one is concerned not with the total number of missed alarms but the number for each fault or some specific faults. Thus the summation in Equation 5 can be replaced by a weighted summation, where the weights correspond to the importance. The weights are difficult to obtain, so we can use the maximization to deal with the bottleneck which is the fault with maximal undetectability. Hence we have the following optimization problem as a combination of a minimaxization and a linear minimization:

(10) $$\underset{x_{j}}{\min}\left[\underset{i}{\max}\left(U_{i}\right)+\alpha \sum_{j=1}^{m}x_{j}V_{j}\right]$$

subject to:

(11) $$\sum_{j=1}^{m}c_{j}x_{j}\leq C_{0},\;x_{j}\in Z^{+}\cup \{0\}$$

If we pay less attention to the false alarm probability, then we can treat it as a constraint and just set a limit *V*_0_. Then we obtain the simplified algorithm:

1. Initialization:
  - Get *f_i_, u_j_* and *v_j_* by a priori knowledge and measurements.
  - Get *d_ij_* from SDG or reachability matrix.
  - Get the minimal *x_j_*s according to detectability and identifiability criteria at the starting point.
  - Calculate *V_j_* by Equation 2.
  - Calculate *V* by summation of all the *V_j_*'s with *x_j_* is not 0.
  - Let the index set of *i*s to be *A*.
2. Calculate *U_i_*.
3. Select the maximal value from *A, U_I_*. If *A* is empty, then stop.
4. Let the set of *j*s with *d_Ij_* is 1 as *A_I_* = {*j*| *d_Ij_*=1}.
5. Select the minimal *u_J_* whose index set is *A_I_*, i.e., *u_J_* = min*_j_*_∈_*_A_I__ u_j_*. If *A_I_* is empty, then delete *I* from *A* and go to step (3). If there is more than one minimum element, select the one with smallest *V_j_*.
6. Place a sensor on variable *J, x_J_* ← *x_J_* + 1.
7. Update the false alarm probability *V*←*V*+ *V_j_*, and see if it is tolerable. If so, then go on; if not, then delete *J* from *A_I_* and go to step (5).
8. Check the cost and other constraints. If they are met, then go on; if not, then delete *J* from *A_I_* and go to step (5).
9. Go to step (2) and update the undetectability.

**Figure 3.**
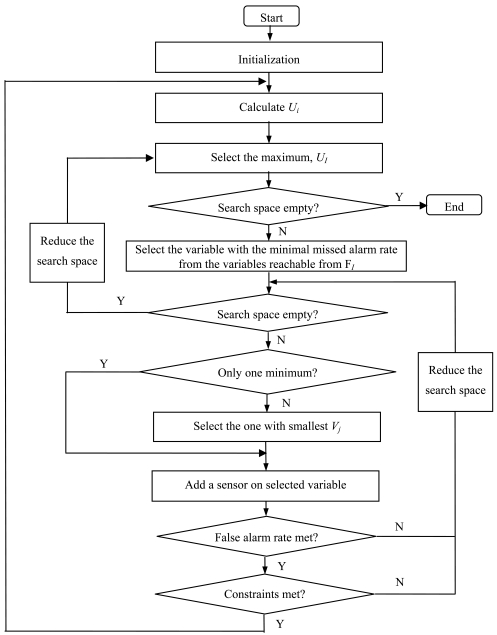
Flow chart of the optimization algorithm.

The algorithm is illustrated as a flow chart in Figure 3.

## 5. Case Study

**Figure 4.**
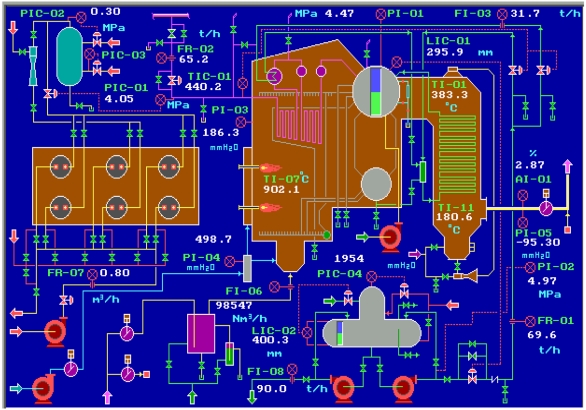
Boiler system flow sheet.

**Figure 5.**
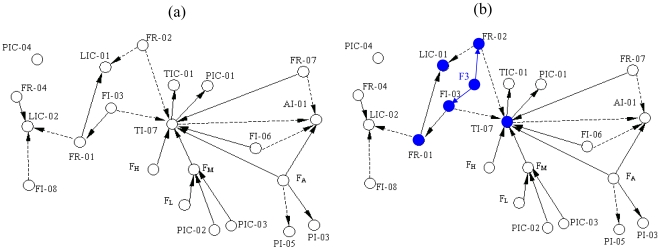
(a) SDG of Boiler system. (b) Fault propagation of fault F3.

We choose a 65 tonnes per hour steam boiler system as an example that is widely used in the power and petrochemical industry, and realize its operation in both normal and abnormal conditions by a simulation software–Personal Simulator [14] whose interface of the main flow chart is shown as Figure 4. The simplified SDG of the system is shown as Figure 5(a) which only describes the relationships between the key variables including inlet flow rate of the boiler FR-01, outlet flow rate of the superheated steam FR-02, flow rate of the cooling water FI-03, flow rate of the softened water FR-04, flow rate of the effluent (smoke) FI-06, flow rate of the fuel oil FR-07, flow rate of the deoxidizing water to be catalyzed FI-08, pressure of the hearth PI-03, pressure of the effluent (smoke) at the exit PI-05, oxygen percentage of the smoke AI-01, pressure of the main steam PIC-01, pressure of the high pressure gas PIC-02, pressure of the liquid hydrocarbon PIC-03, pressure of the deaerator PIC-04, water level of the top steam drum LIC-01, water level of the deaerator LIC-02, temperature of the overheated steam TIC-01, temperature of the hearth TI-07, flow rate of the inlet gas FA, and the flow rate of the high, medium and low pressure gas denoted by FH, FM and FL respectively.

Five typical faults are considered here, all of which are complicated faults that have influences on multiple variables. The faults with their probabilities are listed in Table 1. The missed alarm probability and false alarm probability for all the variables are listed in Table 2 for illustration. In practice, these values are based on the statistics of measurements.

The system's SDG is shown as Figure 5(a) [15] in which the solid lines and dotted lines mean that the arc signs are positive and negative. In the application of this paper, however, the arc signs can be ignored and only the adjacency relations are considered. When the faults occur, some variables become abnormal immediately and then propagate the fault states to other variables. Hence from the SDG, we can easily get the reachability from each fault to each variable. This provides all the possibilities, but because of self-regulatory and control actions, some reachabilities are broken and the corresponding elements remain zeros; we have simulated to validate them. Table 3 shows these reachabilities that are expressed as the transpose of reachability matrix. For example, Fault F3 stands for lack of water in the steam drum, which results in abnormal states on FI-03 and FR-02 directly. With propagation along consistent paths in SDG, several variables such as FR-01, LIC-01 and TI-07 become abnormal as shown in Figure 5(b).

Initially all the variables have sensors except TI-07, FA, FH, FM, FL, which meets the criteria of detectability and identifiability for these five faults. Now we want to reduce the undetectability, so the algorithm we presented is applied. The execution procedure is recorded in Table 4.

By adding two sensors on LIC-01 and FI-03, the maximal undetectability among all the faults reduces 100-fold from 1.5e-4 to 1.5e-6, while the total false alarm probability of all the sensors increases by only 12.9%. In fact, in real systems there are indeed levels of redundancy on the corresponding level sensors and flow meters.

In order to test that the approximation from Equation 7 to 8, the total false alarm probability is computed according to Equation 7. The result is 0.0990, 0.1057 and 0.1087 that are close to the last column of Table 4. Thus the approximation is acceptable.

In this case, the variables that are not affected by any of the five faults can also be ignored in the procedure because placing sensors on them have no influence on the reliability. We can also use other optimization methods to obtain the optimal solution at once if we follow the objective and constraints. We tried this on this example and the results are the same.

## 6. Conclusions

In industrial systems, alarm monitoring design is a very important issue, for which the trade-off between missed alarms and false alarms should be treated appropriately. We should pay attention to two levels of design problems: (1) at the local level, the threshold selection, data filtering and alarm triggering are the key problems to be solved; (2) at the system level, topology expression and sensor location for alarm rationalization is important. In this paper, we have described and solved the sensor location problem aiming at the trade-off with the help of topology expressed by SDG. The optimization objective is expressed as the minimization of all the fault undetectabilities in the system. The false alarm probability is used as constraint as well as the cost limit.

The problem described in this paper is based on some simplifications. For example, the sensors on the same variable are assumed to have the fixed missed alarm probability and false alarm probability. However in reality the sensors can be different and the thresholds are not necessarily the same. So the problem formulation can be generalized as a more accurate form. And the multiple sensors usually do not just add to the redundancy but also provide more information by fusion. Again, future work could be the combination of system level problem and the local level problem.

## Figures and Tables

**Table 1.** Typical faults and their occurrence probabilities in the system.

Faults	Description	Consequences	Probabilities
F2	Steam drum full of water	Inlet reduced heavily	0.1
F3	Lack of water in steam drum	Water level decreases gradually	0.05
F4	Fire extinguishment	All the gas muzzles are extinguished; pressure and temperature of the stream decrease	0.01
F5	Power off	A series of complex phenomenon	0.001
F6	Failure in the cooler	Temperature of overheated steam reduces; cooling water reduces abnormally, etc.	0.001

**Table 2.** Sensor missed alarm probabilities and false alarm probabilities.

Missed alarm probability	False alarm probability	Sensors
0.25	0.002	TIC-01, TI-07, AI-01
0.2	0.003	PI-03, PI-05
0.15	0.004	FR-01, FR-02, FI-03, FR-04, FI-06, FR-07, FI-08
0.08	0.005	FH, FM, FL, FA
0.02	0.008	PIC-01, PIC-02, PIC-03, PIC-04
0.01	0.009	LIC-01, LIC-02

**Table 3.** Reachability from faults to variables.

**Variable**	**F2**	**F3**	**F4**	**F5**	**F6**	***V_j_***
TIC-01	0	0	0	1	1	0.0020
TI-07	0	1	1	1	1	0.0019
AI-01	0	0	1	1	0	0.0020
PI-03	0	0	0	1	0	0.0030
PI-05	0	0	0	1	0	0.0030
FR-01	1	1	1	1	0	0.0034
FR-02	0	1	1	1	0	0.0038
FI-03	0	1	1	1	1	0.0038
FR-04	0	0	1	1	0	0.0040
FI-06	0	0	0	0	0	0.0040
FR-07	0	0	0	1	0	0.0040
FI-08	0	0	0	1	0	0.0040
FH	0	0	0	0	0	0.0050
FM	0	0	0	0	0	0.0050
FL	0	0	0	0	0	0.0050
FA	0	0	0	1	0	0.0050
PIC-01	0	0	1	1	0	0.0109
PIC-02	0	0	0	0	0	0.0080
PIC-03	0	0	0	0	0	0.0080
PIC-04	0	0	0	0	0	0.0080
LIC-01	1	1	1	1	0	0.0076
LIC-02	0	0	0	1	0	0.0090

**Table 4.** Iterative procedure of the algorithm.

**Iteration No.**	***U*_2_ of F2**	***U*_3_ of F3**	***U*_4_ of F4**	***U*_5_ of F5**	***U*_6_ of F6**	**New sensor**	**Total false alarm probability**
0	1.5e-4	1.7e-6	1.3e-8	5.7e-17	3.8e-5		0.0885
1	1.5e-6	1.7e-8	1.3e-10	5.7e-19	3.8e-5	LIC-01	0.0961
2	1.5e-6	2.5e-9	1.9e-11	8.5e-20	5.6e-6	FI-03	0.0999

## Nomenclature

*a*(*i, j*): element of a matrix
*A*: set
*c_i_*: cost to be paid when placing a sensor on variable *j*
*C*_0_: cost limit
*d_ij_*: reachability from fault *i* to sensor *j*
F*_i_*: fault *i*
*F*: fault node
*f_i_*: occurrence probability of fault *i*
*i, I*: serial number of fault
*I*: identifiability set
*j, J*: serial number of sensor
*m*: measureable node, number of measureable nodes
*n*: variable node, number of faults
*R*: reachability set
***R***: reachability matrix
S*j*: sensor *j*
*u_j_*: missed alarm probability of sensor *j*
*U_i_*: undetectability probability of F*i*
*v_i_*: false alarm probability of sensor *j*
*V_i_*: false alarm probability of sensor *j*
*x_j_*: number of sensors placed on variable *j*
***X***: adjacency matrix
